# Cecal MicroRNAome response to *Salmonella enterica* serovar Enteritidis infection in White Leghorn Layer

**DOI:** 10.1186/s12864-016-3413-8

**Published:** 2017-01-13

**Authors:** Guixian Wu, Yukai Qi, Xiaoyi Liu, Ning Yang, Guiyun Xu, Liying Liu, Xianyao Li

**Affiliations:** 1 College of Animal Science and Technology, Shandong Agricultural University, Tai’an, Shandong 271018 China; 2 College of Life Science, Shandong Agricultural University, Tai’an, Shandong 271018 China; 3 College of Animal Science and Technology, China Agricultural University, Beijing, 100193 China; 4 Shandong Provincial Key Laboratory of Animal Biotechnology and Disease Control and Prevention, Tai’an, Shandong 271018 China

**Keywords:** Laying chicken, miRNA, *Salmonella enterica* serovar Enteritidis, Next generation sequencing

## Abstract

**Background:**

*Salmonella enterica* serovar Enteritidis (SE) is a food-borne pathogen and of great threat to human health through consuming the contaminated poultry products. MicroRNAs (miRNAs) play an important role in different biological activities and have been shown to regulate the innate immunity in the bacterial infection. The objective of this study is to identify miRNAs associated with SE infection in laying chicken cecum.

**Results:**

Average number of reads of three libraries constructed from infected and non-infected chickens was 12,476,156 and 10,866,976, respectively. There were 598 miRNAs including 194 potential novel miRNAs identified in which 37 miRNAs were significantly differentially expressed between infected and non-infected chickens. In total, 2897 unique target genes regulated by differentially expressed miRNAs were predicted, in which, 841 genes were uniquely regulated by up-regulated miRNAs (G1), 636 genes were uniquely regulated by down-regulated miRNAs (G2), and 1420 were co-regulated by both up and down- regulated miRNAs (G3). There were 118, 73 and 178 GO (Gene ontology) BP (Biological process) terms significantly enriched in G1, G2 and G3 groups, respectively. More immune-related GO BP terms than metabolism-related terms were found in G1. Expression of 12 immune-related genes of four differentially expressed miRNAs was detected through qRT-PCR. The regulatory direction of gga-miR-1416-5p, gga-miR-1662, and gga-miR-34a-5p were opposite with the target genes of *TLR21*, *BCL10*, *TLR1LA*, *NOTCH2* and *THBS1*, respectively.

**Conclusion:**

The miRNAs contribute to the response to SE infection at the onset of egg laying through regulating the homeostasis between metabolism and immunity. The gga-miR-125b-5p, gga-miR-34a-5p, gga-miR-1416-5p and gga-miR-1662 could play an important role in SE infection through regulating their target genes. The finding herein will pave the foundation for the studies of microRNA regulation in SE infection in laying hens.

**Electronic supplementary material:**

The online version of this article (doi:10.1186/s12864-016-3413-8) contains supplementary material, which is available to authorized users.

## Background

*Salmonellosis* is one of the most important food-borne diseases, with an estimated one million human cases and more than 350 deaths occurring each year in the United States [1]. Non-typhoidal *Salmonella* is the main cause of foodborne disease in the United States [2]. *Salmonella enterica* serotype Enteritidis (SE) is one of the most common serotypes of *Salmonella* bacteria reported worldwide which is the major source of human intestinal infections [3]. The animal or animal product including poultry, eggs, milk and milk products could be the carriers of SE which threaten the human health [4–6]. *Salmonella* organisms could penetrate the intestines mucosal epithelium to outcompete the resident microbial flora and cause the pathological reaction [7]. Egg-related salmonellosis is costing $44 million per year in Australia [8].

MicroRNAs (miRNAs) are small (19 ~ 24 nt long), non-coding, single-stranded and conserved RNAs. It performs multiple functions through regulating gene expression mainly at the post-transcriptional levels [9]. It plays an important role in different biological activities such as the development, cell differentiation and disease [10, 11]. MiRNA could regulate innate immunity caused by viruses, bacteria, fungi, and protozoa infection [12, 13]. MiR-21, miR-146a/b and miR-155 were obviously up-regulated in rat’s mononuclear cells after *Salmonella* infection [14, 15]. Let-7 was down-regulated to induce the release of cytokine IL6 (interleukin 6) and IL10 to participate in the regulation of immune response to *Salmonella* infection in macrophages [14]. MiRNAs also could buffer and alter the variance of relatively lowly expressed genes in the response to *Salmonella* infection in pig [16]. However, the responsive miRNAs in laying hen *Salmonella* infection is still unclear.

The objective of the present study is to discover the miRNAs in the response to *Salmonella* infection in chicken cecum at the onset of egg laying. Next generation sequencing (NGS) has been widely used to analyze the miRNA expression profile in many studies [17–19]. In the current study, the Solexa Sequencing method was used to detect differentially expressed miRNAs in the response to SE infection in White Leghorn at the onset of egg laying. Our results will expand the list of miRNAs related to the host responses to SE infection in chickens.

## Results

### Preliminary analysis of the raw data

Six libraries were constructed from infected (I) and non-infected (N) chickens, three in each group. The average number of total reads of three libraries obtained from I and N chickens were 12,476,156 and 10,866,976, respectively, and the filtered clean reads were 5,078,218 and 2,411,757, respectively (Table 1). In the I group, 3,456,099 clean reads were exactly matched to the chicken genome, 525,400 1-mismatched and 108,131 2-mismatched. In the N group, 1,722,678 clean reads were exactly matched to chicken reference genome, 149,372 1-mismatched and 47,761 2-mismatched. Altogether, 80.5% (4,089,630) and 79.6% (1,919,811) of clean reads were mapped to the genome in the I and N group, respectively. The miRNAs with 21 nt in length were most abundant followed by 22 nt in both I and N groups.

**Table 1:** Number of reads in the infected and non-infected groups

Item	Infected	Non-infected
Total read	12,476,156	10,866,976
Clean data	5,078,218	2,411,757
Exact matched reads	3,456,099	1,722,678
1-mismatch reads	525,400	149,372
2 mismatch reads	108,131	47,761
Total matched reads	4,089,630	1,919,811

**Fig. 1:**
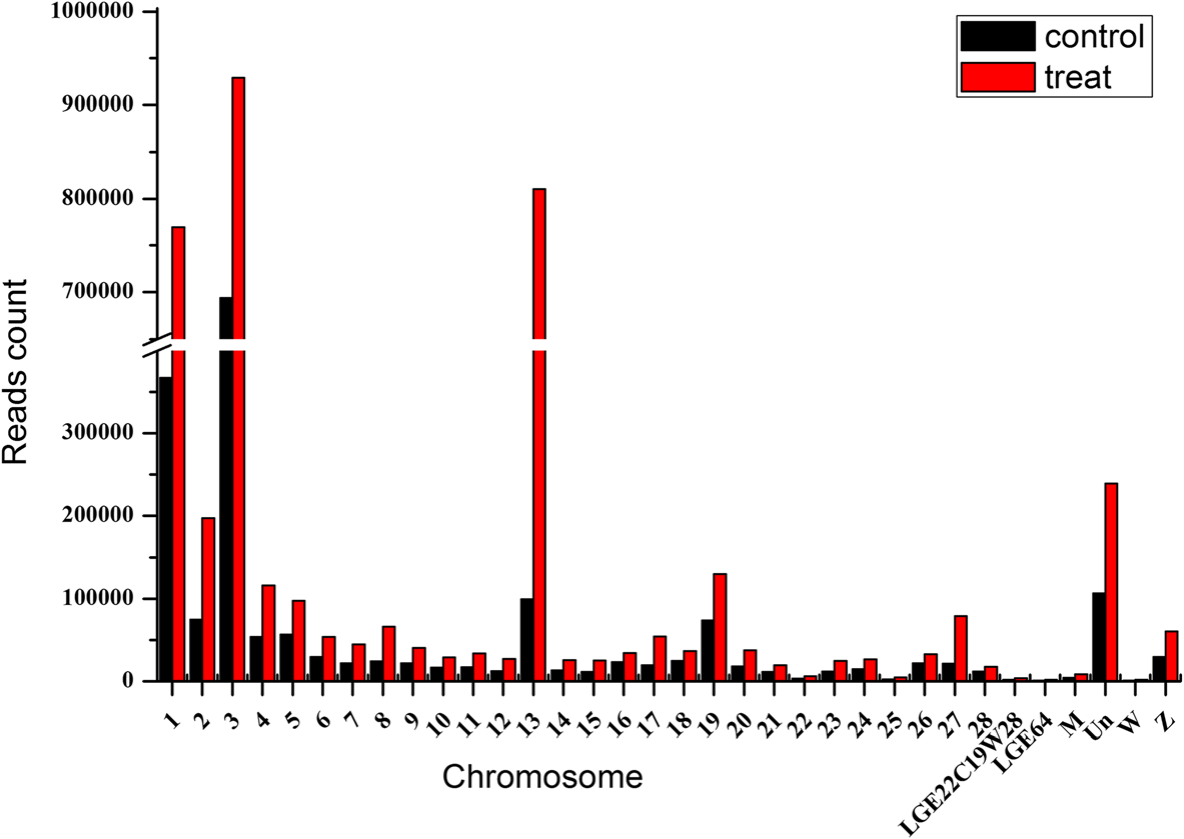
The distribution of clean reads in the genome

Number of reads in each chromosome (Chr) was counted (Additional file 1 and Fig. 1). In general, number of read counts in I group was more than that in N group in each chromosome. For the I group, there were 769,514, 929,207 and 810,295 clean reads matched on Chr1, Chr3, and Chr13, which account for 19.81, 18.82 and 22.72% of total clean reads, respectively. For the N group, there were 367,058 and 693,717 clean reads matched on Chr1 and Chr3, which account for 19.12 and 36.13% of total clean reads, respectively. The count of matched clean reads on Chr13 in I group was eight folds more than that in N group.

### MiRNA identification and genome distribution

**Fig. 2:**
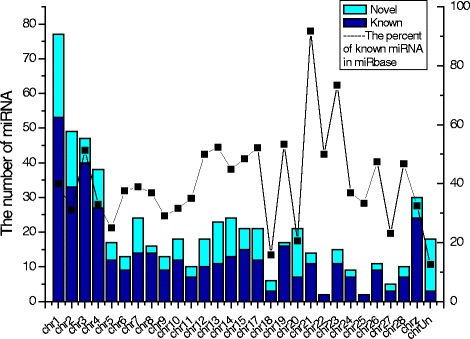
The distribution of miRNAs in genome

To identify the miRNAs, small RNA sequences obtained by deep sequencing were aligned with known miRNA sequences in the miRBase and Rfam (http://pfam.xfam.org). The novel miRNAs were predicted through miRDeep. There were 598 miRNAs including 194 novel miRNAs identified. The number of identified miRNAs in each chromosome was counted (Fig. 2). The chromosomes could be divided into three clusters based on the number of mapped miRNAs: (1) Chr18, Chr22, Chr24, Chr25, Chr27, less than ten miRNAs in each chromosome, (2) Chr5, Chr6, Chr8-12, Chr19, Chr21, Chr23, Chr26 and Chr28, 10–20 miRNAs in each chromosome, (3) Chr1-4, Chr7, Chr13-17, Chr20 and ChrZ, more than 20 miRNAs in each chromosome. There were 77 miRNAs mapped on Chr1 and only two miRNAs on Chr22, respectively. The density distribution of miRNAs across chromosomes showed that number of miRNAs in 1Mbp DNA in each chromosome ranged from 0.19 (ChrW) to 2.77 (Chr21) (Additional file 2). The density of miRNAs in Chr1-12 was lower than one miRNA per 1 Mbp DNA. The density of miRNAs in Chr21, Chr23, Chr26 and Chr28 were more than two miRNAs per 1 Mbp DNA. There were 20–60% of known miRNAs in the miRBase observed in each chromosome except for the Chr21 (91.67%), Chr23 (73.33%), Chr18 (15.79%) and ChrUn (12.5%).

### Differentially expressed miRNAs responding to SE infection

The differentially expressed miRNAs between I and N groups were identified through edgeR package. There were 37 miRNAs significantly differentially expressed between I and N groups including 19 known miRNAs and 18 potentially novel miRNAs (*P* < 0.05 with false discovery rate (FDR) of 0.49 and fold change > 2) (Table 2). There were 22 miRNAs up-regulated post SE infection, which included 15 known and seven novel miRNAs. The highest fold change (71.24) was observed for gga-miR-490- 5p and the lowest fold change (2.06) for gga-miR-193b-3p. Fifteen miRNAs were down-regulated in the comparison of I/N, which included four known and 11 novel miRNAs. The highest fold change (15.13) was observed for gga-miR-chr17_13654 and the lowest (2.01) for gga-miR-chr15_12378. Gga-miR-490-5p, gga-miR-chr13_10137 and gga-miR-chrUn_AADN03024004_45551 were unique to the infected chickens. More than half of significantly differentially expressed miRNAs (17/37) were located on Chr1-10.

**Table 2:** Differentially expressed miRNAs between infected and non-infected chickens (*P* < 0.05, Fold change > 2)

miRNA	Read counts in non-infected	Read counts in infected	Fold change(I/N)	Chromosome position
gga-miR-490-5p	0	52.25	-^a^	chr1:58018956-58019049
gga-miR-chr13_10137	0	21.65	-^a^	chr13:1853152-1853219
gga-miR chrUn_AADN03024004_45551	0	14.83	-^a^	chrUn_AADN03024004:12315-12384
gga-miR-216a	13.32	208.79	15.63	chr3:301576-301682
gga-miR-217-5p	35.76	250.64	6.65	chr3:298908-299015
gga-miR-193a-3p	31.91	153.08	4.27	--------
gga-miR-1a-3p	383.04	1784.26	4.26	chr20:8472264-8472335
gga-miR-1b-3p	298.63	1358.11	4.12	chr23:4294311-4294375
gga-miR-chr6_35375	71.63	300.31	4.05	chr6:2271072-2271138
gga-miR-490-3p	283.61	1123.92	3.76	chr1:58018956-58019049
gga-miR-133a-3p	11115.44	43083.62	3.51	chr2:102176852-102176939
gga-miR-133c-3p	9130.66	33983.52	3.38	chr23:4294450-4294529
gga-miR-133b	8841.86	32748.91	3.36	chr3:107209162-107209246
gga-miR-9-3p	27.29	94.26	3.24	chr28:3378846-3378934
gga-miR-chr13_10219	5157.10	16050.59	2.93	chr13:7833540-7833603
gga-miR-chr13_10222	907675.18	2759302.93	2.86	chr13:7834142-7834200
gga-miR-chr15_12339	221046.01	604996.23	2.59	chr15:463481-463561
gga-miR-chr3_27485	334.10	844.81	2.41	chr3:65480575-65480649
gga-miR-1416-5p	177.32	420.27	2.30	chrZ:34781500-34781589
gga-miR-125b-3p	1026.62	2508.62	2.29	chr1:98380667-98380757
gga-miR-125b-5p	6220.19	14048.45	2.10	chr1:98380667-98380757
gga-miR-193b-3p	216.46	492.77	2.06	chr14:762080-762163
gga-miR-chr15_12378	10551.58	5882.44	−2.01	chr15:4870074-4870145
gga-miR-215-5p	6638307.42	3618379.46	−2.02	chr3:18143688-18143793
gga-miR-147	3180.31	1712.26	−2.10	chr10:10170160-10170230
gga-miR-1662	2111.61	1000.82	−2.39	chr2:1818924-1818997
gga-miR-chr7_36925	1908.29	858.02	−2.50	chr7:6845145-6845212
gga-miR-chr2_16700	141.08	49.79	−2.78	chr2:73136006-73136063
gga-miR-34a-5p	278.17	99.89	−2.93	chr21:3266631-3266740
gga-miR-chr10_6644	27.94	3.81	−3.85	chr10:7087285-7087353
gga-miR-chr1_5477	36.18	4.44	−4.69	chr1:152779416-152779543
gga-miR-chr10_6507	51.26	13.08	−4.88	chr10:1689882-1689969
gga-miR-chrUn_AADN03024004_45550	16.72	1.27	−5.56	chrUn_AADN03024004:1006-1069
gga-miR-chr20_19772	62.48	14.47	−6.10	chr20:10132999-10133045
gga-miR-chr20_19953	281.37	24.37	−15.02	chr20:1049342-1049435
gga-miR-chr20_19955	281.37	24.37	−15.02	chr20:1153806-1153899
gga-miR-chr17_13654	40.24	1.27	−15.13	chr17:6752602-6752677

^a^Specifically expressed in infected group

**Fig. 3:**
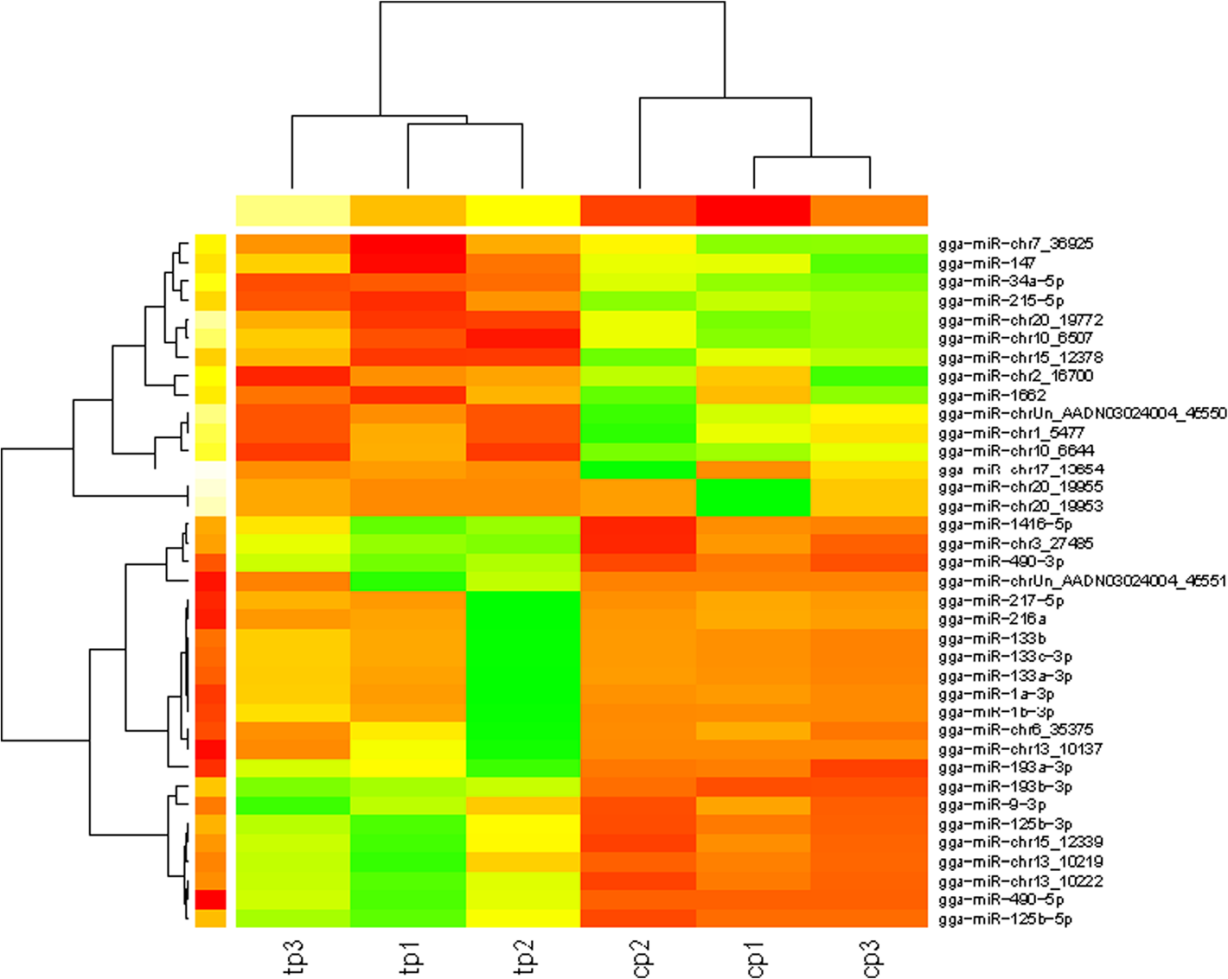
The heatmap of the differentially expressed miRNAs. Note: The heat map was computed using a function of heatmap.2 in gplots by R platform. The green indicated higher miRNA expression level and the red showed lower miRNA expression level. Tp1, tp2 and tp3 were the sample pools in the I group, cp1, cp2 and cp2 were sample pools in the N group

The heat map and hierarchical clustering demonstrated that the miRNA profiles from I and N groups were distinct (Fig. 3). The infected and non-infected groups were separated distinctly. The differentially expressed miRNAs were clustered into two groups. MiRNAs in group one showed higher expression in the N group but lower expression in I group, which including gga-miR-34a-5p, gga-miR-215-5p, and gga-miR-1662. MiRNAs in group two showed higher expression in I group but lower expression in N group including gga-miR-125b-3p, gga-miR-1416-5p and gga-miR-125b-5p.

### MiRNA target gene identification

To investigate the biological role of the differentially expressed miRNAs, the potential target genes were predicted using Miranda algorithm with the Vienna package. In total, 2897 unique target genes regulated by those differentially expressed miRNAs were predicted, in which, 841 genes were uniquely regulated by up-regulated miRNAs (G1), 636 genes were uniquely regulated by down-regulated miRNAs (G2), and 1420 were co-regulated by both up and down-regulated miRNAs (G3). One hundred and seventy-six immune-related genes were retrieved from the Ensembl BioMart database [20]. In total, 587 pairs of interaction between miRNAs and immune-related genes were obtained (Additional file 3). Gga-miR-34a-5p interacted with the greatest number of immune-related genes (46), while the gga-miR-215-5p only interacted with one immune-related gene.

### Gene ontology (GO) analysis for target genes

**Fig. 4:**
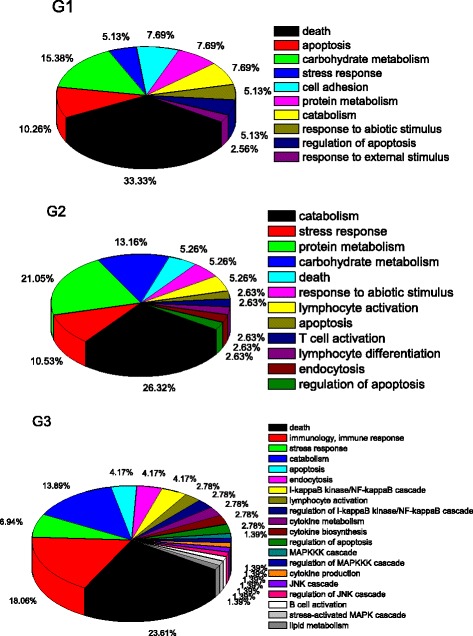
The classification of GO BP terms associated with target genes. Note: G1, genes uniquely regulated by up-regulated miRNAs; G2, genes uniquely regulated by down-regulated miRNAs; G3, genes co-regulated by both up and down-regulated miRNAs

To demonstrate the function of target genes of differentially expressed miRNAs, functional annotation was performed through DAVID (The Database for Annotation, Visualization and Integrated Discovery) for the target genes in the G1, G2 and G3 groups, respectively (Additional file 4). There were 118, 73 and 178 GO BP (biological process) terms significantly enriched in G1, G2 and G3, respectively (*P* < 0.05). The GO BP terms were categorized by CateGOrizer using “Immune System Gene Classes” GO classification (Fig. 4). For target genes in G1, the enriched GO BP terms were categorized into ten ancestral classes, which could be divided into three groups: 1) immune-related function including death, apoptosis, cell adhesion, stress response, response to abiotic stimulus, regulation of apoptosis, and response to external stimulus occupied 61.54% of all ancestral classes, 2) metabolism-related including carbohydrate metabolism, protein metabolism, and catabolism, occupied 30.76% of all ancestral classes, 3) cell adhesion occupied 7.69% of all ancestral classes. For target genes in G2, the enriched GO BP terms were categorized into 12 ancestral classes. The immune-related classes including stress response, death, response to abiotic stimulus, lymphocyte activation, apoptosis, T cell activation, lymphocyte differentiation, regulation of apoptosis, occupied 36.83% of all ancestral classes. The metabolism-related classes including catabolism, protein metabolism, carbohydrate metabolism, occupied 60.53% of all ancestral classes. For target genes in G3, the enriched BP terms were categorized into 20 ancestral classes, the immune-related classes occupied 80.58% of all ancestral classes, and metabolism-related classes occupied 15.28%.

### KEGG Pathway analysis

For the target genes in the G1, only one pathway of endocytosis was significantly enriched (*P* < 0.05) with the fold enrichment of 1.65, which included 18 target genes. For target genes in the G2, five pathways were significantly enriched, which were ECM-receptor interaction, glycolysis/gluconeogenesis, focal adhesion, melanogenesis and proteasome. Proteasome had the highest fold enrichment of 3.53 and focal adhesion included the largest number of target genes (16). For target genes in the G3, Cell cycle and SNARE interactions in vesicular transport were significantly enriched and associated with 27 and 9 genes, respectively (Table 3).

**Table 3:** The significantly enriched pathways in three groups of target genes (*P* < 0.05)

	Pathway	No. of genes	*P* value	Fold enrichment	Genes
G1	Endocytosis	18	0.040	1.65	CHMP2A, HRAS, CLTC, CHMP2B, EPS15, SMAP2, AP2A2, TFRC, RABEP1, CXCR4, WWP1, NTRK1, MDM2, IL2RG, PDCD6IP, EGF, EHD3, AP2M1
G2	ECM-receptor interaction	10	0.007	2.86	CD47, COL4A2, ITGA8, ITGAV, COL6A3, ITGA3, VTN, SDC2, SDC3, SPP1
	Glycolysis/Gluconeogenesis	7	0.017	3.32	LDHB, GPI, TPI1, LDHA, PGM1, DLD, PGAM1
	Focal adhesion	16	0.018	1.91	COL4A2, MAP2K1, ACTN1, ITGA3, VTN, PPP1CC, CDC42, CCND1, ITGA8, ITGAV, COL6A3, ILK, RHOA, MAPK9, FIGF, SPP1
	Melanogenesis	10	0.020	2.43	WNT5A, KRAS, MAP2K1, CALM, MAP2K2, ADCY5, FZD1, CAMK2D, WNT6, FZD7
	Proteasome	6	0.024	3.53	PSMC3, PSMA4, PSMC2, PSMD3, PSME3, PSMD4
G3	Cell cycle	27	3.68E-04	2.04	E2F1, YWHAZ, E2F4, E2F5, ANAPC10, CIP1, CDKN2B, TFDP2, BUB1, CCNA2, RBL2, YWHAB, SKP2, RB1, SKP1, CDC27, MCM5, ATM, CDC25A, WEE1, CDK3, YWHAG, YWHAH, CCND3, HDAC1, PLK1, YWHAQ
	SNARE interactions in vesicular transport	9	0.041	2.21	STX6, STX17, USE1, BET1L, SEC22B, VAMP3, GOSR2, SNAP23, GOSR1

*Note*: G1 means the unique target genes of up-regulated miRNAs, G2 means the unique target genes of down-regulated miRNAs, G3 means the target genes regulated by both up and down-regulated miRNAs

### Protein-protein Interacting Network of the immune-related target genes

**Fig. 5:**
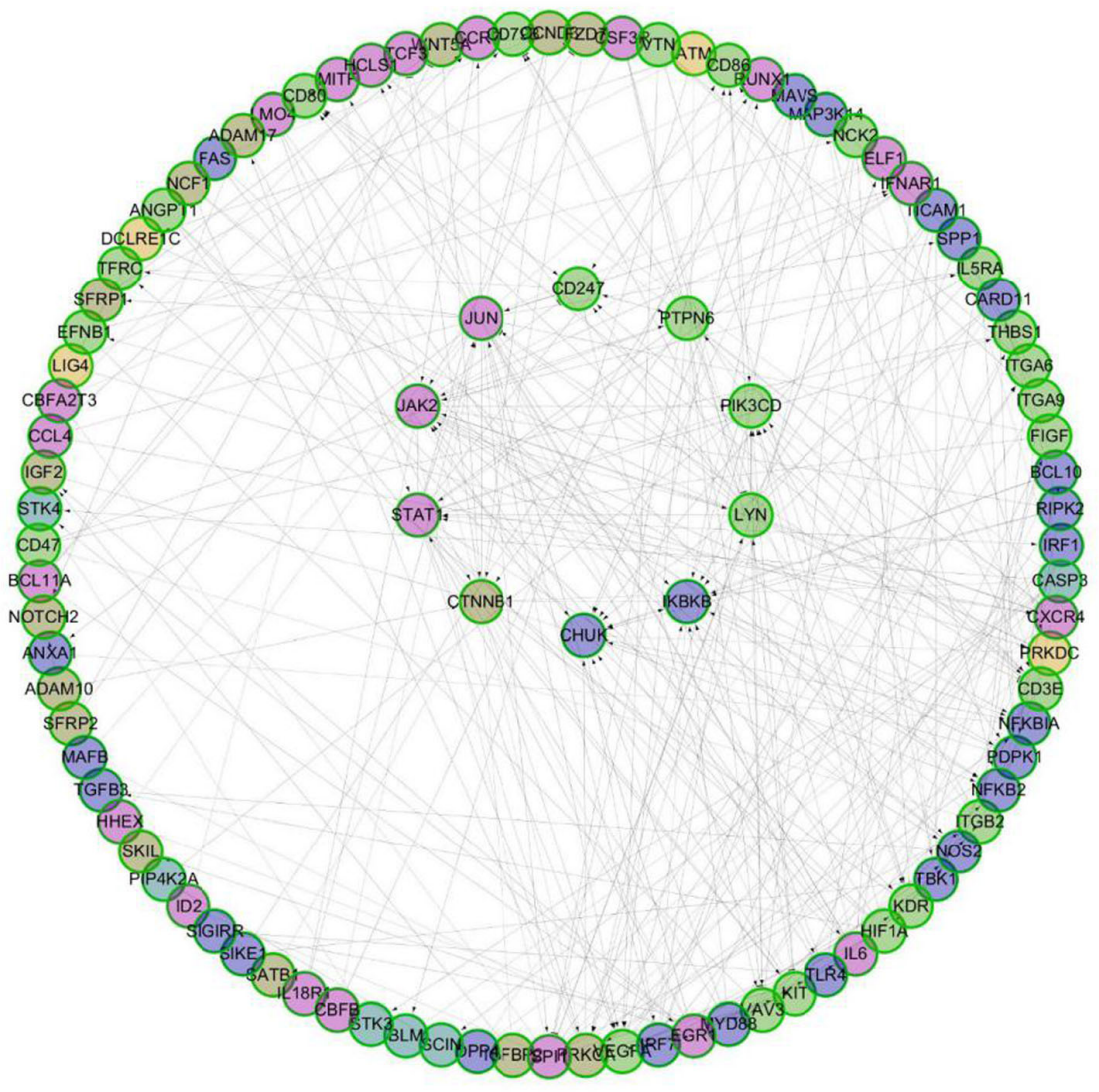
Protein-protein interaction network of the immune-related target genes

The Reactome FIViz (http://f1000research.com/articles/3-146/v2) app in Cytoscape was used to examine the potential protein-protein interactions of the 176 immune-related genes encoded proteins [21]. Through Cytoscape, there were 98 proteins accepted by Reactome FIViz and clustered into six network modules (Fig. 5). The top ten proteins interacted with more than 15 other proteins were illustrated in the center of the network. JUN protein interacted with 32 proteins, and LYN and JAK with 21 proteins, respectively.

### Validation of differentially expressed miRNAs

The relative expression level of 12 differentially expressed miRNAs regulating immune-related genes was validated through quantitative real-time PCR (qRT-PCR). The specific primers for those 12 miRNAs (seven up-regulated miRNAs (including two novel miRNAs) and five down-regulated miRNAs (including two novel miRNAs)) were listed in Additional file 5. The results showed that all the miRNAs selected for qRT-PCR validation were significantly differentially expressed. All the results in qRT-PCR were consistent with those obtained from the sequencing except for gga-miR-193b-3p which had reverse regulatory direction (Table 4). The gga-miR-1416-5p and gga-miR-125b-5p were up-regulated. The gga-miR-1662 and gga-miR-34a-5p were down-regulated after SE infection.

**Table 4:** Fold change of miRNAs obtained from qRT-PCR and Solexa Sequencing

miRNA	qRT-PCR	Sequencing
gga-miR-34a-5p	0.363*	0.342*
gga-miR-chr15_12378	0.382**	0.498*
gga-miR-chr7_36925	0.274**	0.400*
gga-miR-1662	0.418**	0.419*
gga-miR-215-5p	0.416**	0.495*
gga-miR-133b	2.283**	3.359*
gga-miR-chr13_10219	2.229**	2.931**
gga-miR-chr13_10222	1.810*	2.862**
gga-miR-1b-3p	2.808**	4.115**
gga-miR-1416-5p	2.602**	2.297*
gga-miR-193b-3p	0.575*	2.060*
gga-miR-125b-5p	2.335**	2.095*

*Note*: *, *P* < 0.05; **, *P* < 0.01

### Target genes expression

**Fig. 6:**
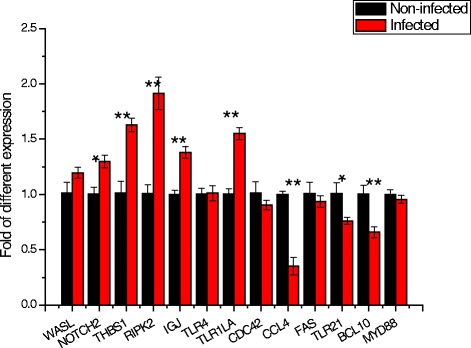
Qualification of immune-related target genes of differentially expressed miRNAs

Twelve immune-related target genes of 4 differentially expressed miRNAs (gga-miR-1416-5p, gga-miR-1662, gga-miR-125b-5p and gga-miR-34a-5p) were selected to detect the relative expression using qRT-PCR. The potential regulation was listed in Additional file 6. The specific primers were listed in Additional file 7. The results showed that eight target genes were significantly differentially expressed between I and N groups (Fig. 6). *NOTCH2*, *THBS1*, *RIPK2*, *IGJ* and *TLR1LA* were significantly up-regulated (*P* < 0.05) and *CCL4*, *TLR21* and *BCL10* were significantly down-regulated following SE infection. Five of eight target genes had reversely regulatory direction with their regulating miRNAs following SE infection.

## Discussion

MiRNAs play a vital role in regulating immunity induced by bacteria [22–24]. Regulation of miRNA expression responding to bacterial infection is emerging as a crucial part of the host response to infection [22]. The onset of egg laying is a complex physiological process when physiological and hormonal stress occurs. Previously, we have studied the splenic gene expression in laying hens through microarray [25]. In the current study, cecal miRNA profiling in the response to SE infection in laying hens has been identified through Solexa sequencing. In total, 404 known miRNAs and 194 potential novel miRNAs were expressed in six libraries.

MiRNA distribution varies widely in different chromosome in different species, and chromosomes with higher number of miRNAs most likely play regulatory roles in several cellular processes including disease. MiRNAs associated with cancer and cardiovascular disease are mostly located in Chr1, 14, 19, and X in human [26]. There is a significant association between the chromosomal location of miRNAs and those of cancer susceptibility loci in mouse [27]. There are 39.66% miRNA genes located on chicken chromosome 1, 2, 3, and 4 [26]. The skew distribution of miRNAs has been observed following SE infection in the current study. Majority of miRNAs responding to SE infection located on Chr1, 2, 3, 10, 13, and 20. There was no miRNAs observed in chicken Chr16 which is consistent with the current results from miRBase database (http://www.mirbase.org).

SE infection stimulates the miRNAs expression. Thirty-seven miRNAs were differentially expressed following SE infection. Read counts in the infected group were higher than those in the non-infected group (Fig. 1). In the current study, more up-regulated miRNAs were observed at 7 days post infection (dpi) (22 up-regulated vs. 15 down-regulated). More miRNAs are up-regulated at 2 days post infection with *S. Typhimurium* in piglet which is consistent with current miRNA profiling [28]. The similar response to Gram-negative bacterial infection could be regulated by miRNAs. MiRNA regulation in immune response is influenced by genetic background. It has been reported that opposite miRNA profiling was found between SPF layer and broiler chickens infected with AIV (Avian Influenza Virus) [19, 29]. Functionally, the miRNAs target mRNAs and trigger either translation repression or RNA degradation in animal [30]. It has been reported that the complicated interaction between immune system and metabolism exists during SE infection in laying hens [31]. Maintaining metabolic homeostasis requires a balanced immune response. The perturbation of this equilibrium could result in pathological situations [32]. It has been reported that miRNAs are fine-tuners of metabolic processes [33] and could generate a bidirectional functional link between metabolism and pathogenesis [32]. In the current study, target genes of up-regulated miRNAs were associated with stronger immune function than metabolism function. Target genes of down-regulated miRNAs were associated with stronger metabolism compared with immune function. The target genes regulated by both up- and down-regulated miRNAs were also associated with stronger immune function than metabolism (Fig. 4). The target genes of up- and down regulated miRNAs may buffer the homeostasis between immune response and metabolism. Those miRNAs may contribute to buffer the gene expression of target genes [16, 32]. The immune function was repressed at the onset of egg laying [25, 34, 35]. The results herein suggested that miRNAs could regulate the interaction between immune response and metabolism on day 7 post SE infection in laying hens.

It is noteworthy that the Proteasome pathway had the highest fold enrichment in the enriched pathways. The proteasome is a protein-destroying apparatus involved in many essential cellular functions including antigen processing for appropriate immune responses and inflammatory responses [36–38]. The nonclassical class Ib molecule Qa-1b is a dominant restricting element to the recognition of CD8+ T Cells which is proteasome-dependent during *Salmonella* infection [39]. Type III protein secretion systems (T3SS) effector protein activities of *Salmonella* are temporally regulated by proteasome-dependent protein degradation [40]. The LMP2 protein, a subunit of the cytosolic proteasome complex, is up-regulated after invasion of HLA-B27-transfected HeLa cells by *Salmonella typhimurium* [41]. Both *Salmonella enteritidis* and *Salmonella typhimurium* are gram-negative bacteria. This could suggest that proteasome is an important defense system to protect against bacterial colonization regulated by miRNAs in chicken.

MiR-34a-5p was down-regulated and potentially targeted 46 immune-related genes in chicken after SE infection. MiR-34a was associated with the cellular senescence, it was strongly up-regulated and targeted the important proto-oncogene *MYC* during B-RAF-induced senescence [42]. MiR-34a may play an important role in the loss of oxidative defense in rat liver during aging through suppressing the expression of Sirt1, Mgst1, Sp1 and Nrf2 [43]. *Bcl-2* and the *Foxp1* transcription factor, which were required for early B cell development [44], are the direct target of miR-34a, so it was hypothesized that miR-34a plays an important role in B cell development [45–47]. MiR-34a causes a partial block in B cell development, whereas its knockdown results in increased B cells development in rat bone marrow [48]. *CCL4*, *IL8L1* and *CDC42* were contained in the *Salmonella* infection pathway and were all targeted by miR-34a. *CCL4* gene is significantly up-regulated at different time points (1, 2, 4, and 8 h) after SE endotoxin treatment [49]. *CDC42* could activate the Arp2/3 by N-WASP (Neural Wiskott-Aldrich syndrome protein) to mediate actin polymerization in the invasion of SE [50–53]. It has been reported that mir-34a exclusively expressed in AIV infected chicken lung with targeting 14 immune-related genes and four AIV genes [29]. Although there were no SE genes regulated by mir-34a predicted, the down-regulation of miR-34a may conduce to B cell development in the response to SE infection in chickens.

Gga-miR-1416 is located in the intron of MAMDC 2 (MAM domain containing 2) on ChrZ. Gga-miR-1416 is up-regulated in both layer and broiler following AIV infection suggests that it responds to AIV infection across diverse genetic lines [29]. Six immune-related genes were potentially targeted by gga-miR-1416-5p including *BCL10*, *NFKBIA* and *TLR21*, which were important in the response to bacterial infection. *BCL10* mediates the LPS-induced activation of *NF-κB* and *IL8* in normal human intestinal epithelial cells [54]. Chicken *TLR21* is involved in the recognition of bacterial components and *Salmonella* in the innate immune response [55]. The expression of *TLR 21* was decreased in the duodenum, jejunum, ileum, ceca and large intestine of broilers following SE infection [56]. In the current study, the expression of *TLR 21* was significantly down-regulated and had reversely regulatory direction with the regulating gga-miR-1416-5p post SE infection. Gga-miR-1416-5p could play an important role in response to SE infection through regulating the target genes.

The interaction of miRNA with target genes plays an important role in many biological functions. Toll-like receptors (*TLR*) recognizes specific patterns of microbial components and participate in the innate immunity and antigen-specific adaptive immunity [57]. As one of the main pattern recognition receptors, *TLR* can identify the pathogen to activate the immune cells response and induce to produce type I interferon and a series of proinflammatory cytokines [58]. *TLR1LA* could combine with *TLR2* to form a dimer and efficiently identify bacterial peptidoglycan and lipoprotein [59]. *TLR1LA* is significantly up-regulated at the ileum, cecum and colon at 24 h post SE infection in day-old chickens [56]. Gga-miR-1662 was significantly up-regulated in chicken lung following AIV infection [29]. Gga-miR-1662 was down-regulated while its target gene *TLR1LA* was up-regulated in the response to SE infection in the current study. The interaction of gga-miR-1662 with *TLR1LA* could be important in the respond to SE infection in chicken. Further study is warranted to verify the function of interaction between gga-miR-1662 and *TLR1LA*.

## Conclusions

MiRNAs mediate the homeostasis between metabolism and immunity in the response to SE infection at the onset of egg laying. The gga-miR-34a-5p, gga-miR-1416-5p and gga-miR-1662 could play important roles in SE infection through regulating their target genes. The finding herein will pave the foundation for the studies of microRNA regulation in SE infection in laying hens.

## Methods

### Animals and SE challenges

One hundred 20-week old SE negative White Leghorn layers were used in the current study. Chickens were randomly divided into two groups with equal number, infected group (I) and non-infected (N) group. At the first day, chickens in the infected group were fed with 30 g feed containing 5.8 × 10^8^ cfu SE at a time and the non-infected birds were fed with regular feed as described previously (Wu et al., [25]). Chickens in each group were sacrificed by cervical dislocation at 7 days post infection (dpi). The cecum samples were aseptically harvested from each chicken and put into RNAlater solution (Life technologies, Grand Island, USA) and stored at –20 °C. All animal procedures were approved by Shandong Agricultural University Animal Care and Use Committee.

### Small RNAs library construction and deep sequencing

In total, nine infected and six non-infected birds were randomly selected for further RNA isolation. Total RNA was isolated from each individual sample using Trizol following the manufacture’s protocol (Life technologies, Grand Island, USA). The RNA integrity and concentration were checked and measured using gel electrophoresis and Nanodrop (Thermo Fisher Scientific, Wilmington, USA).

Six small RNA libraries were pooled from I and N groups, three in each group. Each small RNA library from I (tp1, tp2, tp3) and N (cp1, cp2, cp3) group consisted of three and two individual samples with equal amount of RNA, respectively. The small RNA pools were purified and enriched using denaturing polyacrylamide gel electrophoresis. A pair of Illumina proprietary adaptors was ligated to their 5’ and 3’ ends, followed by reverse transcription and cluster generation using TruSeq Small RNA Library Preparation Kit (Illumina Inc., San Diego, USA). Subsequently, the libraries were sequenced by Illumina Hiseq 2500 according to the manufacturer’s instructions at Genergy Inc (Shanghai, China).

### Basic data processing

The small RNA sequence reads were pre-processed using FASTX-Toolkit to filter low-quality reads and trim adaptor. After filtering adaptor sequences and removing contaminated reads, the clean reads were matched to chicken reference genome using the Bowtie [60]. The first 15 bp of the read was exactly matched. Two mismatched bases were accepted on the rest nucleotides of the read. The filtered sequences were matched with miRBase (http://mirbase.org/) to search for known miRNAs with exact matches. The unmatched data sets were aligned with chicken genomic sequence (Gallus_gallus-4.0) and predict the novel miRNAs using miRDeep [61].

### Differential expression analysis of miRNAs

To compare miRNAs expression level between I and N groups, read count of each identified miRNA was normalized to the total number of reads in each given sample. The differentially expressed miRNAs were identified through edgeR package with False discovery rate multiple testing correction [62]. The *P* < 0.05 and fold change > 2 was considered as significant difference.

### Target prediction and Gene Ontology (GO) enrichment analysis

The target genes of those significantly differentially expressed miRNAs were predicted using Miranda algorithm through the Vienna package [63, 64]. Functional annotation of GO and pathway analysis for those target genes of miRNAs were performed through DAVID 6.7 [65–67]. Significant over-representation is based on a Fisher Exact statistical methodology similar to that described by Al-Shahrour et al [68]. CateGOrizer was used to categorize the significantly enriched GO BP (biological process) terms [69].

### Protein-protein Interaction Network Analysis of the immune-related target genes

We converted the gene IDs of 176 immune-related target genes to the gene symbols of the encoded proteins using bioDBnet software (www.biodbnet.abcc.ncifcrf.gov/db/db2 db.php#biodb). Then the gene symbols were mapped onto the chicken functional interaction network found in the Reactome database using the Reactome FI network plug-in in the Cytoscape software [70].

### Quantitative real-time PCR of miRNAs and target genes

The same individual RNA samples used for sequencing were used for quantitative real-time PCR (qRT-PCR). In total, nine infected and six non-infected samples were used. Twelve differentially expressed miRNAs were validated and characterized using qRT-PCR. In brief, 1 μg of total RNA was reverse transcribed using Step PrimeScript® miRNA cDNA Synthesis Kit (Perfect Real Time) and amplified using Stratagene MX3000 real-time PCR System with miRNA specific primers (Additional file 5). The qRT-PCR was performed with SYBR green PCR master mix. Small nucleolar RNA U6 was used as endogenous control to normalize RNA input. The 20 μL PCR reactions included 10 μL SYBR® Premix Ex TaqTM II (2×), 0.4 μL ROX Reference Dye (50×), 0.8 μL Uni-miR qPCR Primer (10 μM), 0.8 μL forward qPCR Primer, 2 μL cDNA and 6 μL ddH_2_O. The qPCR amplification conditions were: 1 cycle of 95 °C for 30s, 40 cycles of 95 °C for 5 s and 60 °C for 30s. All qRT-PCR reactions were performed in triplicate. The relative expression were calculated using 2^-△△CT^ method. The student’s T-test was performed to examine the significance of miRNA expression between I and N groups.

Relative expression level of 12 immune-related target genes were quantified using qRT-PCR. The RNA was reverse transcribed to cDNA using TaKaRa Primer Script™ RT reagent kit (Perfect Real Time) (TaKaRa, Dalian, China) according to the manufacturer’s manual. The qRT-PCR was performed by Stratagene MX3000 real-time PCR System with SYBR green method. The specific primers were designed by primer premier 5.0 according to gene sequence (Additional file 7). The 20 μL PCR reactions included 10 μl SYBR Primer Ex Taq^TM^ (2×), 0.4 μl forward primer (10 μM), 0.4 μl reverse primer (10 μM), 0.4 μL ROX Reference Dye II (50×), 2 μL cDNA, 6.8 μL ddH_2_O. The conditions of qRT-PCR amplification and data analysis were the same as those used in miRNA qRT-PCR. The chicken β-actin was used as the internal control.

## Additional files

Additional file 1: The distribution of clean reads in the genome. (DOCX 13 kb)
Additional file 2: Density distribution of miRNAs across chromosomes. Note: Densities are shown as number of miRNAs per megabase of DNA. (TIFF 256 kb)
Additional file 3: Immune-related target genes and interaction with miRNAs. (XLS 128 kb)
Additional file 4: Enriched GO BP terms in each group. (XLS 100 kb)
Additional file 5: Primer information for miRNAs validated by qRT-PCR. (DOCX 17 kb)
Additional file 6: The potential regulation between miRNAs and target genes. (DOCX 15 kb)
Additional file 7: The designed primers for genes validated by qRT-PCR. (DOC 43 kb)

## Abbreviations

SE: *Salmonella enterica* serovar Enteritidis
miRNA: MicroRNA
GO: Gene ontology
BP: Biological process
G1: Genes were uniquely regulated by up-regulated miRNAs
G1: Genes were uniquely regulated by down-regulated miRNAs
G3: Genes co-regulated by both up and down- regulated miRNAs
NGS: Next generation sequencing
I: Infected
N: Non-infected
Chr: Chromosome; ChrUn contains clone contigs that can’t be confidently placed on a specific chromosome
DAVID: The Database for Annotation, Visualization and Integrated Discovery
KEGG: Kyoto Encyclopedia of Genes and Genomes
qRT-PCR: Quantitative real-time PCR
AIV: Avian influenza virus
TTSS: Type III protein secretion systems
TLR: Toll like receptor
IL: Interleukin
FDR: False discovery rate
