# Supplementary material for: Cecal MicroRNAome response to Salmonella enterica serovar Enteritidis infection in White Leghorn Layer
Source: BMC Genomics. 2017 Jan 13;18:77. doi: 10.1186/s12864-016-3413-8 (PMC5237128; doi:10.1186/s12864-016-3413-8)
Supplement: Additional file 1: — The distribution of clean reads in the genome. (DOCX 13 kb) [file 12864_2016_3413_MOESM1_ESM.docx]

**Additional file 1**

**Table s1**

**The distribution of clean reads in the genome**

|  | infected | | Non-infected | |
| --- | --- | --- | --- | --- |
|  | The average reads count | percentage | The average reads count | percentage |
| chr1 | 769514 | 18.82% | 367058 | 19.12% |
| chr2 | 197306 | 4.82% | 74807 | 3.90% |
| chr3 | 929207 | 22.72% | 693717 | 36.13% |
| chr4 | 116382 | 2.85% | 54105 | 2.82% |
| chr5 | 97911 | 2.39% | 56906 | 2.96% |
| chr6 | 54070 | 1.32% | 29644 | 1.54% |
| chr7 | 44894 | 1.10% | 22073 | 1.15% |
| chr8 | 66111 | 1.62% | 24458 | 1.27% |
| chr9 | 40385 | 0.99% | 22156 | 1.15% |
| chr10 | 29245 | 0.72% | 16558 | 0.86% |
| chr11 | 34085 | 0.83% | 17338 | 0.90% |
| chr12 | 27161 | 0.66% | 12445 | 0.65% |
| chr13 | 810295 | 19.81% | 99487 | 5.18% |
| chr14 | 25806 | 0.63% | 13477 | 0.70% |
| chr15 | 25358 | 0.62% | 11318 | 0.59% |
| chr16 | 34171 | 0.84% | 23220 | 1.21% |
| chr17 | 54406 | 1.33% | 19874 | 1.04% |
| chr18 | 36917 | 0.90% | 24887 | 1.30% |
| chr19 | 130330 | 3.19% | 73968 | 3.85% |
| chr20 | 37618 | 0.92% | 18012 | 0.94% |
| chr21 | 19435 | 0.48% | 11365 | 0.59% |
| chr22 | 6080 | 0.15% | 3316 | 0.17% |
| chr23 | 24692 | 0.60% | 12093 | 0.63% |
| chr24 | 26612 | 0.65% | 15052 | 0.78% |
| chr25 | 5068 | 0.12% | 2505 | 0.13% |
| chr26 | 33107 | 0.81% | 21850 | 1.14% |
| chr27 | 79112 | 1.93% | 21657 | 1.13% |
| chr28 | 17792 | 0.44% | 11821 | 0.62% |
| chrLGE22C19W28 | 3934 | 0.10% | 2197 | 0.11% |
| chrLGE64 | 2091 | 0.05% | 999 | 0.05% |
| chrM | 8898 | 0.22% | 4521 | 0.24% |
| chrUn | 239269 | 5.85% | 106617 | 5.55% |
| chrW | 1989 | 0.05% | 901 | 0.05% |
| chrZ | 60380 | 1.48% | 29411 | 1.53% |
| total | 4089630 | 100.00% | 1919811 | 100.00% |
