# Supplementary figures and images for: Cecal MicroRNAome response to Salmonella enterica serovar Enteritidis infection in White Leghorn Layer
Source: BMC Genomics. 2017 Jan 13;18:77. doi: 10.1186/s12864-016-3413-8 (PMC5237128; doi:10.1186/s12864-016-3413-8)

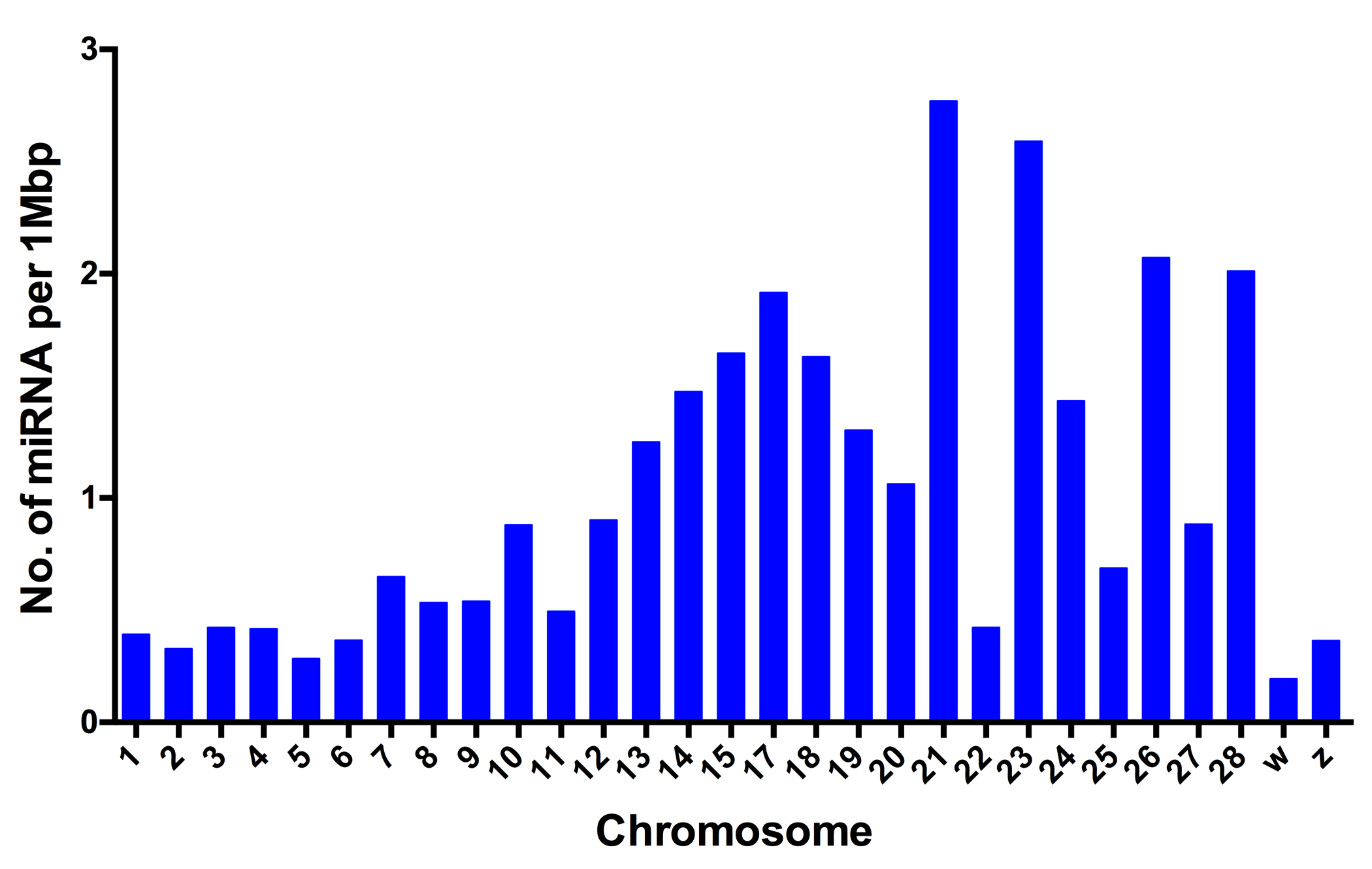

Supplement: Additional file 2: — Density distribution of miRNAs across chromosomes. Note: Densities are shown as number of miRNAs per megabase of DNA. (TIFF 256 kb) [file 12864_2016_3413_MOESM2_ESM.tiff]
