# Supplementary material for: Cecal MicroRNAome response to Salmonella enterica serovar Enteritidis infection in White Leghorn Layer
Source: BMC Genomics. 2017 Jan 13;18:77. doi: 10.1186/s12864-016-3413-8 (PMC5237128; doi:10.1186/s12864-016-3413-8)
Supplement: Additional file 5: — Primer information for miRNAs validated by qRT-PCR. (DOCX 17 kb) [file 12864_2016_3413_MOESM5_ESM.docx]

**Additional file 5**

**Primer information for miRNAs validated by QRT-PCR**

| Primer | Sequences (5′→3′) | Length (nt) | GC (%) | Tm |
| --- | --- | --- | --- | --- |
| >gga-miR-125b-5p | TCCCTGAGACCCTAACTTGTGA | 22 | 50.0 | 60.1 |
| >gga-miR-133b | TACGTCCCCTTCAACCAGCTA | 21 | 52.4 | 60.0 |
| >gga-miR-193b-3p | GCCCACCAAGTCCCGCTTT | 19 | 63.2 | 61.9 |
| >gga-miR-chr13_10219 | GGCCAGTTGTCCCATGAATCCCT | 23 | 56.5 | 63.7 |
| >gga-miR-chr13_10222 | TGAGATGATGCACTGTAGCTCG | 22 | 50.0 | 60.1 |
| >gga-miR-34a-5p | TGTCAGTGTCTTAGCTGGTTGTT | 23 | 43.5 | 58.4 |
| >gga-miR-chr15_12378 | GGGGATGTAGCTCAGTGGTAGA | 22 | 54.5 | 61.9 |
| >gga-miR-chr7_36925 | CGCGTCTAGGGGTATGATTCTC | 22 | 54.5 | 61.9 |
| >gga-miR-1b-3p | GGCGGTGGAATGTTAAGAAGTATGTA | 26 | 42.3 | 60.4 |
| >gga-miR-1416-5p | TCCTTAACTCATGCCGCTGTG | 21 | 52.4 | 60.0 |
| >gga-miR-1662 | CGTGGACATCATCATACTTGGGAT | 24 | 45.8 | 60.3 |
| >gga-miR-215-5p | GCCGATGACCTATGAATTGACAGAC | 25 | 48.0 | 62.0 |
| U6 | GGCCAAGGATGACACGCAAA | 20 | 55.0 | 61.0 |
