# Supplementary material for: Cecal MicroRNAome response to Salmonella enterica serovar Enteritidis infection in White Leghorn Layer
Source: BMC Genomics. 2017 Jan 13;18:77. doi: 10.1186/s12864-016-3413-8 (PMC5237128; doi:10.1186/s12864-016-3413-8)
Supplement: Additional file 6: — The potential regulation between miRNAs and target genes. (DOCX 15 kb) [file 12864_2016_3413_MOESM6_ESM.docx]

**Additional file 6**

**The potential regulation between miRNAs and target genes**

| miRNA | Target genes |
| --- | --- |
| gga-miR-1416-5p | IGJ |
|  | TLR21 |
|  | BCL10 |
| gga-miR-1662 | MYD88 |
|  | TLR1LA |
| gga-miR-125b-5p | WASL |
|  | RIPK2 |
|  | FAS |
| gga-miR-34a-5p | NOTCH |
|  | THBS1 |
|  | CDC42 |
|  | CCL4 |
