# Supplementary material for: Cecal MicroRNAome response to Salmonella enterica serovar Enteritidis infection in White Leghorn Layer
Source: BMC Genomics. 2017 Jan 13;18:77. doi: 10.1186/s12864-016-3413-8 (PMC5237128; doi:10.1186/s12864-016-3413-8)
Supplement: Additional file 7: — The designed primers for genes validated by qRT-PCR. (DOC 43 kb) [file 12864_2016_3413_MOESM7_ESM.doc]

**Additional file 7**

**The designed primers for gene validated by QRT-PCR**

| Gene | Accession No. | Sequence (5'-3') | |
| --- | --- | --- | --- |
| MYD88 | NM_001030962 | forward | TTACGAAGGAAGCAGCAGGA |
|  |  | reverse | CTGACAGTAGCAGATGAAGGCA |
| TLR1LA | NM_001007488 | forward | GCTTGACTTTAGTGCCTTCATGTTT |
|  |  | reverse | GCAAGCAATTGGCAGTAAGCT |
| WASL | XM_415994 | forward | AAAGGACAATCCGCAGAGGT |
|  |  | reverse | TCTCCAGCGAAGGTATGAAAAT |
| CDC42 | NM_205048 | forward | GTGATGATTGGAGGAGAGCC |
|  |  | reverse | TGAAGAAGGAGATACCACTGAAA |
| CCL4 | NM_204720 | forward | CCTCGCTGTCCTCCTCATT |
|  |  | reverse | CACTGGCTGTTGGTCTCGT |
| NOTCH2 | XM_001233595 | forward | ATTGATGATTGCTTCACGGC |
|  |  | reverse | TCTGACACGGATTGCTAACACA |
| THBS1 | XM_421205 | forward | GGACATTCCCATCCAAAACA |
|  |  | reverse | AAACACAAACCGCACATTCTG |
| FAS | XM_421659 | forward | TTACAGTTTCAGTGGTCAGTGCT |
|  |  | reverse | CATCTCATACACTCGTCCAAATC |
| RIPK2 | NM_001030943 | forward | AGCGAAACTTCCTTGCCAG |
|  |  | reverse | TTCCTTGACATCACTTCCCATA |
| TLR21 | NM_001030558 | forward | GATGGAGACAGCGGAGAA |
|  |  | reverse | GCGGAAGTACAAAGGTGC |
| IGJ | NM_204263 | forward | TGGGATGATGGTGAGGAGC |
|  |  | reverse | TGCGGTAGACAAAAGTGGTTC |
| BCL10 | XM_422365 | forward | GGACTGGATGCTTTGGTTGA |
|  |  | reverse | GAAGGGAGACAGCAGACATAAAT |
| β-actin | NM_205518 | forward | TGCTGTGTTCCCATCTATCG |
|  |  | reverse | TTGGTGACAATACCGTGTTCA |
